# Supplementary material for: Assessing Biodegradability of Chemical Compounds from Microbial Community Growth Using Flow Cytometry
Source: mSystems. 2021 Feb 9;6(1):e01143-20. doi: 10.1128/mSystems.01143-20 (PMC7883543; doi:10.1128/mSystems.01143-20)
Supplement: TABLE S1 [file mSystems.01143-20-st001.docx]

| **Experiment objective** | **Test compounds** | **Carbon dosage**  **(mg C l^–1^ )** | **Initial cell density  (cells ml^–1^)** | **Inoculum sampling date** |
| --- | --- | --- | --- | --- |
| Effects of different concentrations  (High Concentrations) | Benzoate, Phenol, 1-Octanol | 0.1, 1 , 10 , 100 , 1000 | 10^5^ | 05.09.2017 |
| Effects of different concentrations  (Fragrances) | Methyl jasmonate | 0.1, 1, 10 | 10^5^ | 12.05.2017 |
|  | Myrcene | 0.1, 1, 10 | 10^5^ | 24.05.2017 |
| Effects of starting density and different concentrations  (Initial Conditions) | Benzoate | 0.1, 0.5 , 1 , 2 , 10 | 10^4^, 10^5^, 10^6^ | 11.05.2016 |
|  | Phenol | 0.1, 0.5 , 1 , 2 , 10 | 10^4^, 10^5^, 10^6^ | 06.06.2016 |
|  | 1-Octanol | 0.1, 0.5 , 1 , 2 , 10 | 10^4^, 10^5^, 10^6^ | 18.07.2016 |
| Mass balance analysis | 1-Octanol | 10 | 10^5^ | 24.04.2017 |
|  | Phenol | 10 | 10^5^ | 06.06.2017 |
| Mass balance analysis (Fragrances) | Methyl jasmonate | 10 | 10^5^ | 25.09.2017 |
|  | Myrcene | 10 | 10^5^ | 12.02.2018 |
|  | Musk-xylene | 10 | 10^5^ | 25.09.2017 |
| ^14^C mass balance analysis | ^14^C-1-Octanol | 0.1, 1, 10 | 10^5^ | 13.11.2018 |
|  | ^14^C-Phenol | 0.1, 1, 10 | 10^5^ | 17.10.2017 13.11.2017 |
| 16S rRNA amplicon community analysis | Phenol, 1-octanol, methyl jasmonate, myrcene | 10 | 10^5^ | 08.01.2019 |
| Effect of dead cells | Phenol | 10 | 10^6^ | 10.12.2019 |
