# Supplementary material for: Assessing Biodegradability of Chemical Compounds from Microbial Community Growth Using Flow Cytometry
Source: mSystems. 2021 Feb 9;6(1):e01143-20. doi: 10.1128/mSystems.01143-20 (PMC7883543; doi:10.1128/mSystems.01143-20)
Supplement: TABLE S2 [file mSystems.01143-20-st002.docx]

| **Compound** | **Concentration (mg l^–1^)** | | |
| --- | --- | --- | --- |
|  | **Lake Geneva**  **Water** ^a^ | **ALW mineral medium** | **OECD mineral medium** ^b^ |
| Calcium (Ca) | 45.4 | 8.5 | 8.51 |
| Magnesium (Mg) | 5.8 | 11.1 | 2.22 |
| Nitrogen (N) | NA | 8.7 | 3.38 |
| Nitrates (NO_3_-N) | 2.7 | 4.3 | 1.21 |
| Sulfates (SO_4_) | 46.4 | 43.8 | 2.93 |
| Phosphorus (P) | NA | 17.4 | 116.14 |
| Potassium (K) | 1.7 | 24.4 | 122.07 |
| Sodium (Na) | 9.1 | 8.6 | 86.28 |
| Chlorides (Cl) | 11.5 | 19.3 | 22.77 |
| Iron (Fe) | 0.002 | 0.04 | 0.04 |
| Zinc (Zn) | <0.001 | - | - |
| Copper (Cu) | 0.001 | - | - |

^a^ <http://www.sig-ge.ch/en/ecoinfo/use-less-save-more/drink-eau-de-geneve/characteristics-and-benefits/composition-of-water>

^b^ OECD, 2014
