# Supplementary figures and images for: Assessing Biodegradability of Chemical Compounds from Microbial Community Growth Using Flow Cytometry
Source: mSystems. 2021 Feb 9;6(1):e01143-20. doi: 10.1128/mSystems.01143-20 (PMC7883543; doi:10.1128/mSystems.01143-20)

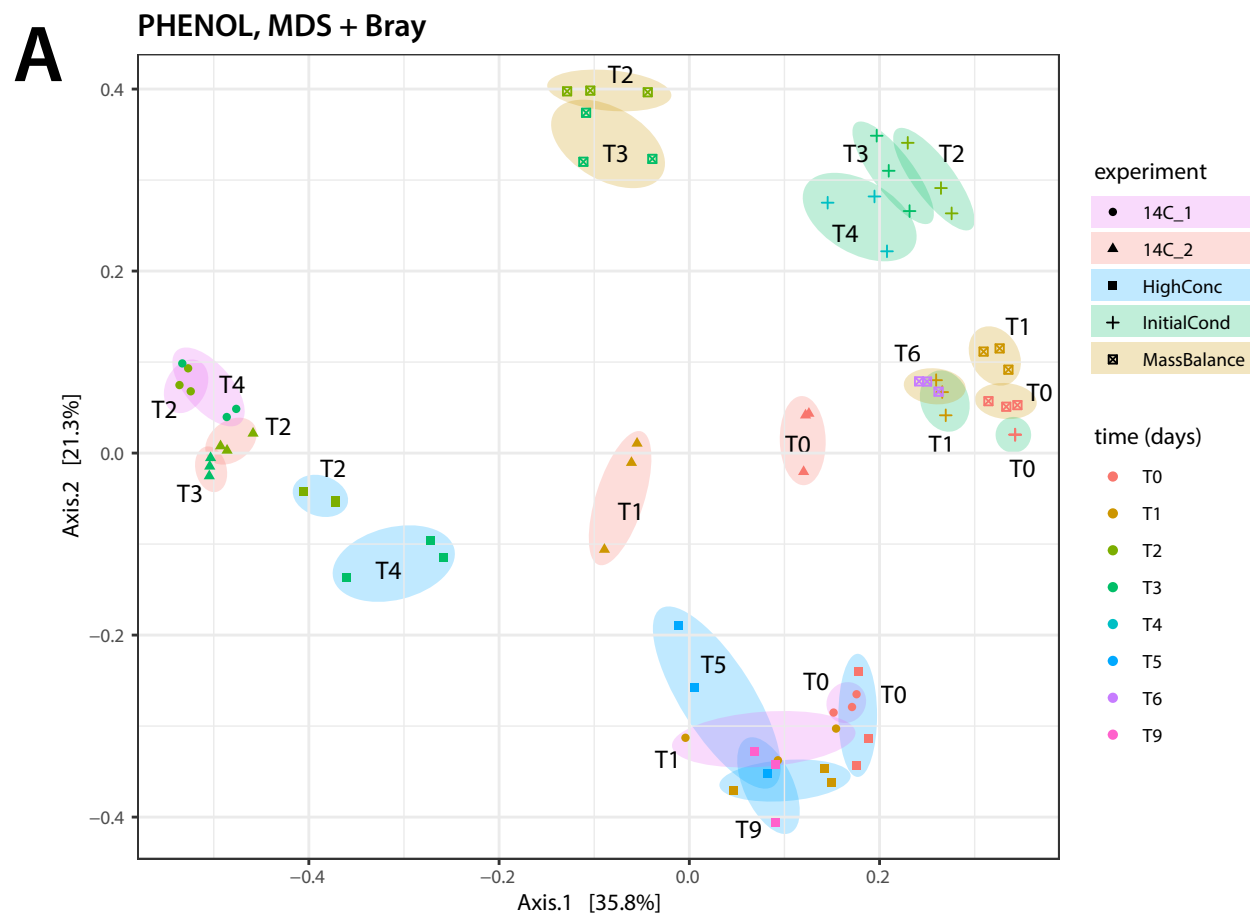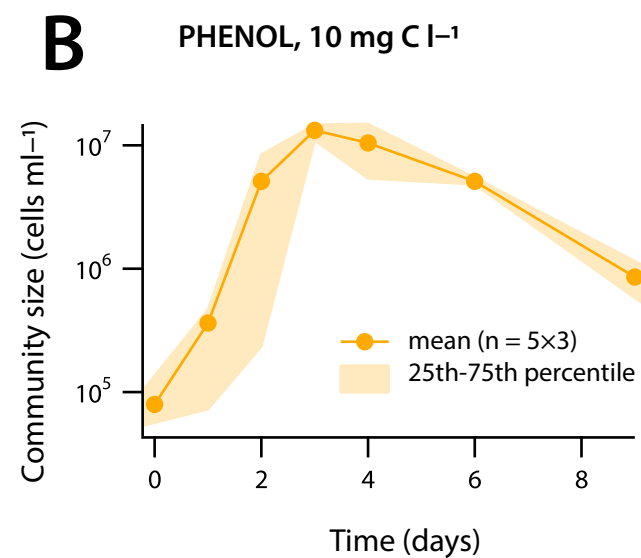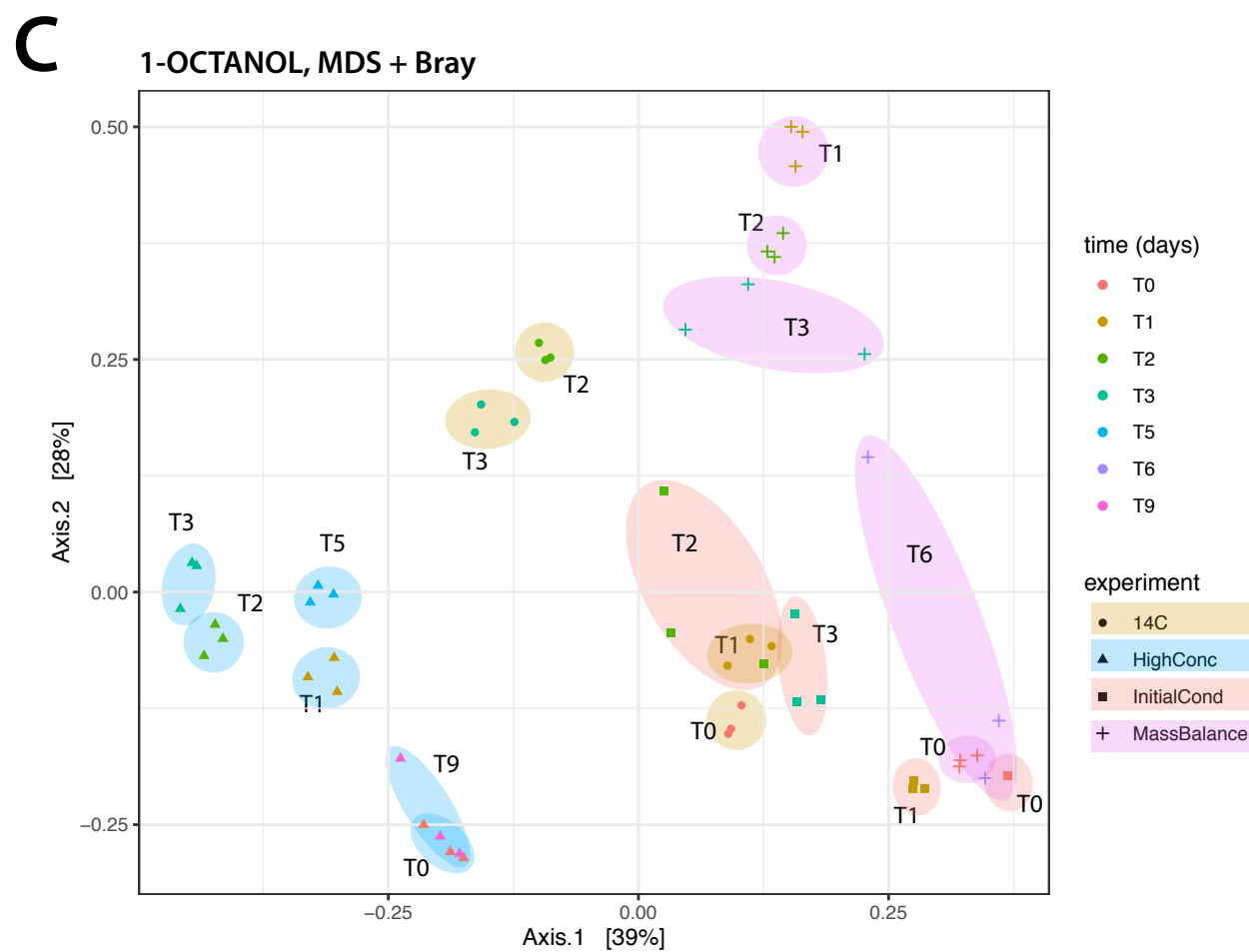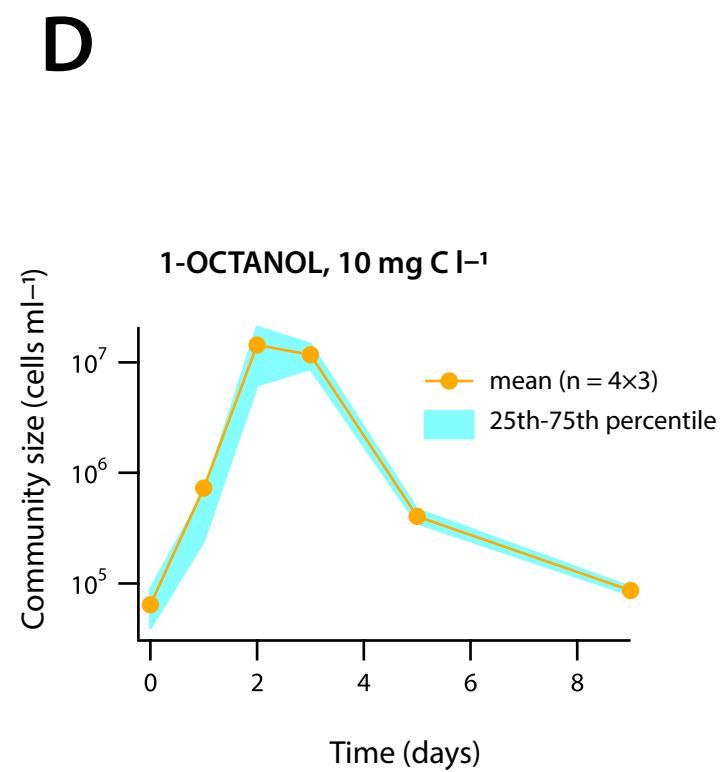

Supplement: FIG S1 [file mSystems.01143-20-sf001.pdf]

**A****Phenol (exp 1)**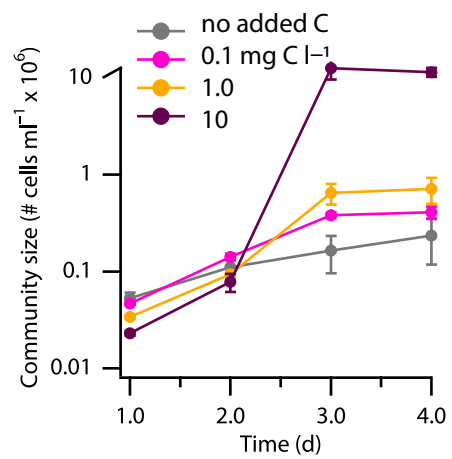**B****Phenol (exp 2)**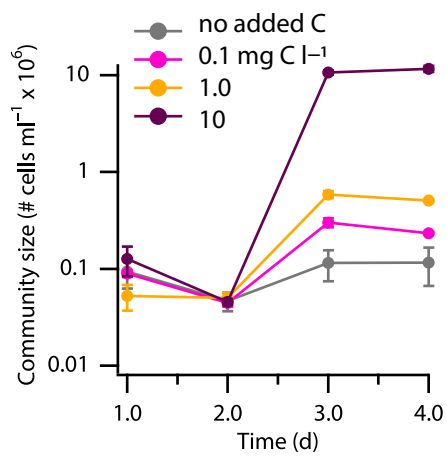**C****1-Octanol**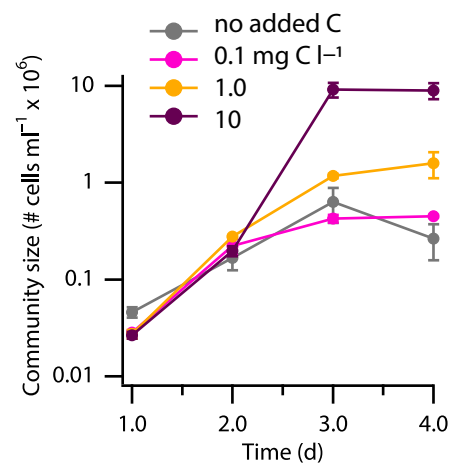

Supplement: FIG S2 [file mSystems.01143-20-sf002.pdf]

A

PHENOL, MDS + Bray

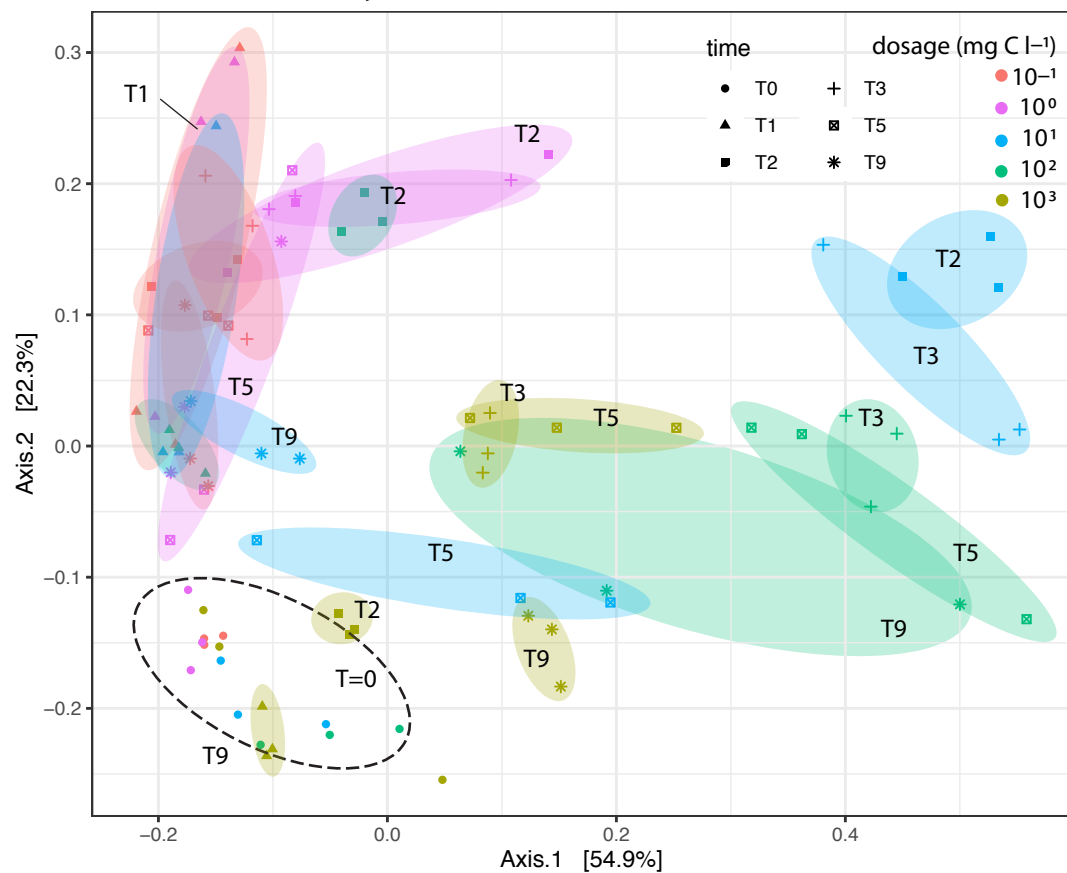

B

1-OCTANOL, MDS + Bray

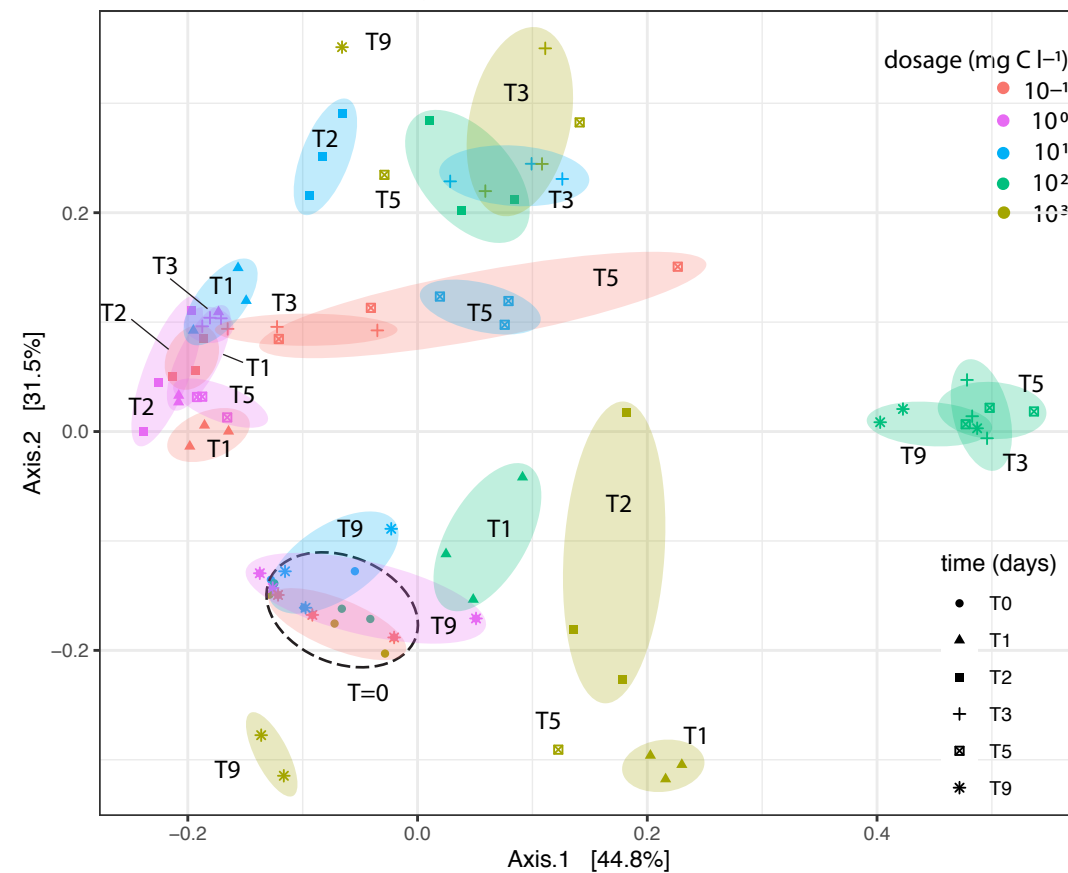

Supplement: FIG S3 [file mSystems.01143-20-sf003.pdf]

**A**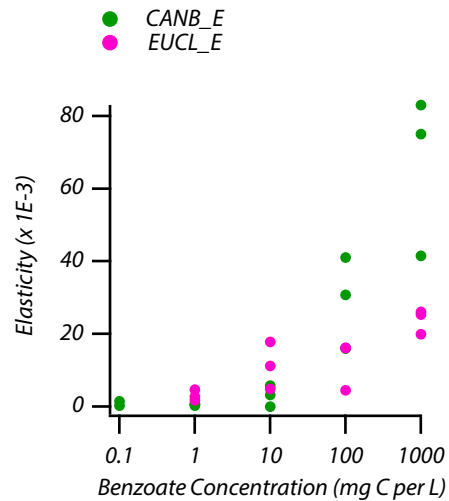**B**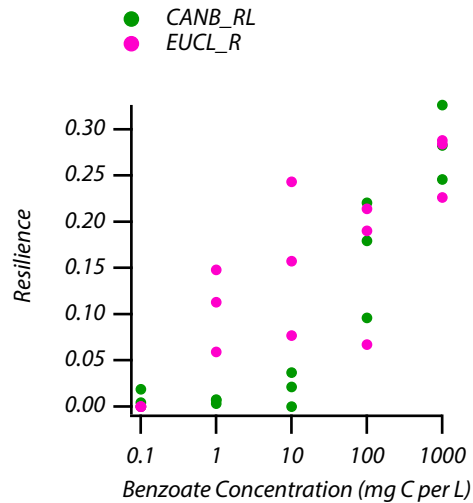

Supplement: FIG S4 [file mSystems.01143-20-sf004.pdf]

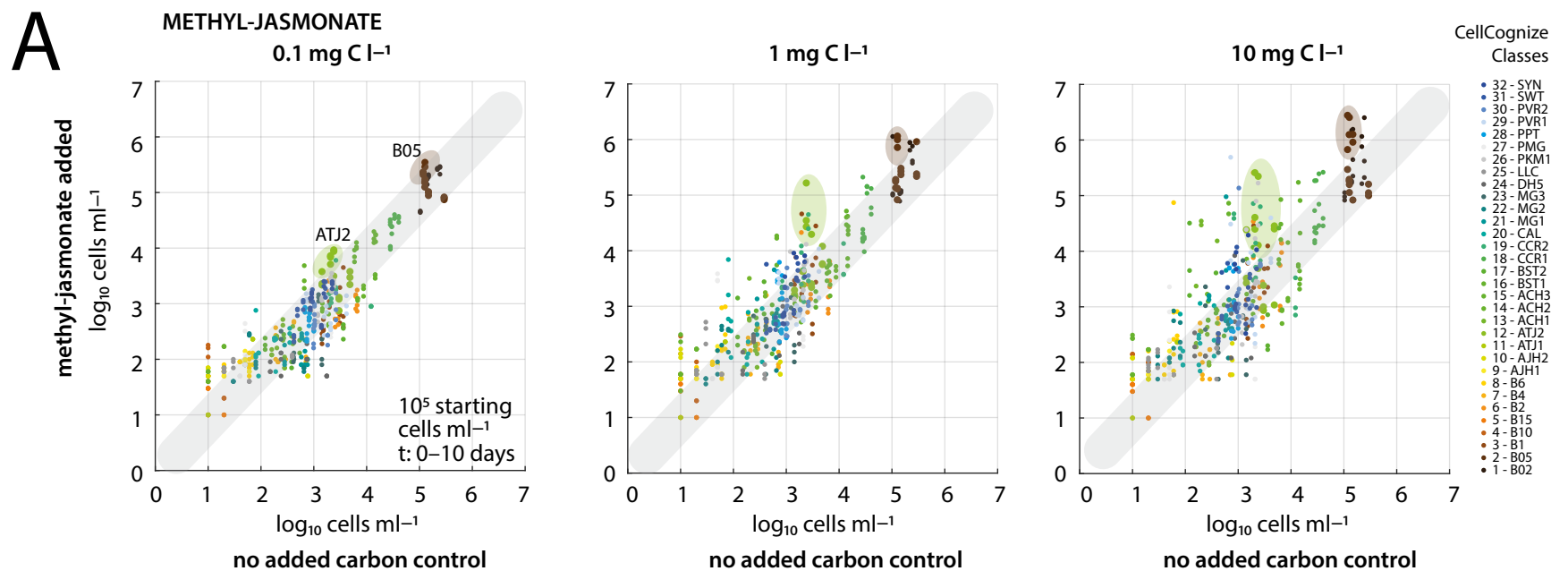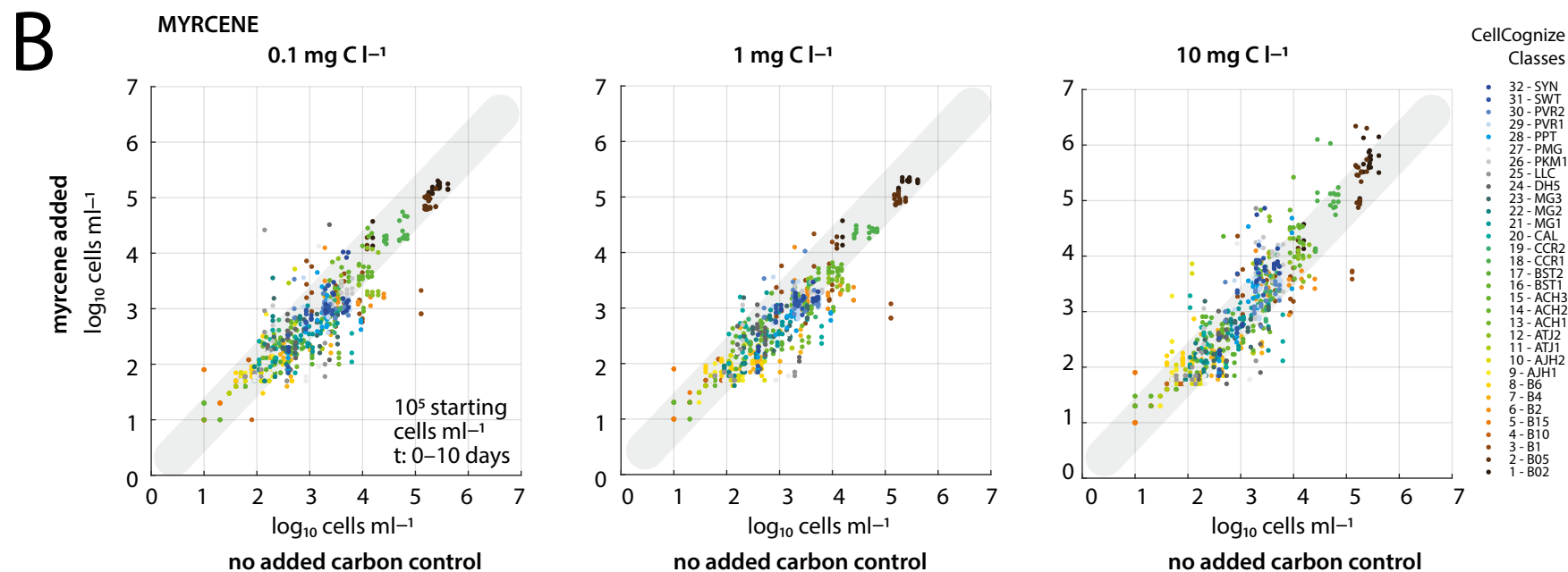

Supplement: FIG S5 [file mSystems.01143-20-sf005.pdf]
