# Supplementary material for: Assessing Biodegradability of Chemical Compounds from Microbial Community Growth Using Flow Cytometry
Source: mSystems. 2021 Feb 9;6(1):e01143-20. doi: 10.1128/mSystems.01143-20 (PMC7883543; doi:10.1128/mSystems.01143-20)
Supplement: TABLE S3 [file mSystems.01143-20-st003.docx]

| index nr | FDR | Q-value | | fold change to ALW | | mean read count (per 10^5^) in substrate amended sample | | mean read count (per 10^5^) in ALW medium | | OTU assignment (at 99% to SILVA) | |  |
| --- | --- | --- | --- | --- | --- | --- | --- | --- | --- | --- | --- | --- |
| PHENOL, T = 3 days | | | | |  | |  | |  | |  | |
| 27 | 0.009 | 0.008 | | Inf | | 153.495 | | 0.000 | | Holophagales;D_4__Holophagaceae;D_5__uncultured | |  |
| 38 | 0.008 | 0.008 | | Inf | | 70.858 | | 0.000 | | Acidobacteria;D_2_subgroup_5;_ | |  |
| 51 | 0.008 | 0.008 | | Inf | | 141.716 | | 0.000 | | Microtrichales;D_4__Iamiaceae;D_5__Iamia | |  |
| 63 | 0.000 | 0.000 | | 28.198 | | 10507.523 | | 372.632 | | Bifidobacteriales;D_4__Bifidobacteriaceae;D_5__Bifidobacterium | |  |
| 213 | 0.004 | 0.004 | | 10.361 | | 2223.130 | | 214.558 | | Cytophagales;D_4__Spirosomaceae;D_5__Emticicia | |  |
| 217 | 0.001 | 0.001 | | 13.727 | | 7152.142 | | 521.036 | | Cytophagales;D_4__Spirosomaceae;D_5__Pseudarcicella | |  |
| 223 | 0.000 | 0.000 | | 23.679 | | 29927.366 | | 1263.884 | | Flavobacteriales;D_4__Crocinitomicaceae;D_5__Fluviicola | |  |
| 224 | 0.001 | 0.001 | | 11.251 | | 6003.344 | | 533.562 | | Flavobacteriales;D_4__Crocinitomicaceae;D_5__uncultured | |  |
| 229 | 0.000 | 0.000 | | 14.956 | | 60496.505 | | 4044.992 | | Flavobacteriales;D_4__Flavobacteriaceae;D_5__Flavobacterium | |  |
| 234 | 0.023 | 0.023 | | 10.414 | | 306.990 | | 29.479 | | Flavobacteriales;D_4__NS9 marine group;D_5__uncultured bacterium | |  |
| 238 | 0.009 | 0.008 | | Inf | | 177.145 | | 0.000 | | Flavobacteriales;D_4__Weeksellaceae;D_5__Elizabethkingia | |  |
| 260 | 0.001 | 0.001 | | 34.586 | | 13901.393 | | 401.941 | | Sphingobacteriales;D_4__env.OPS 17;D_5__uncultured bacterium | |  |
| 261 | 0.001 | 0.001 | | 101.608 | | 16740.792 | | 164.759 | | Sphingobacteriales;D_4__env.OPS 17;__ | |  |
| 603 | 0.022 | 0.022 | | 13.885 | | 409.320 | | 29.479 | | Gracillibacteria;_;_ | |  |
| 667 | 0.003 | 0.003 | | 10.504 | | 821.531 | | 78.215 | | Acetobacterales;D_4__Acetobacteraceae;D_5__Roseomonas | |  |
| 788 | 0.008 | 0.008 | | 20.172 | | 3214.653 | | 159.363 | | Sphingomonadales;D_4__Sphingomonadaceae;D_5__Sphingopyxis | |  |
| 903 | 0.000 | 0.000 | | 61.548 | | 72006.746 | | 1169.919 | | Betaproteobacteriales;D_4__Burkholderiaceae;D_5__Acidovorax | |  |
| 906 | 0.000 | 0.000 | | Inf | | 23702.953 | | 0.000 | | Betaproteobacteriales;D_4__Burkholderiaceae;D_5__Comamonas | |  |
| 907 | 0.000 | 0.000 | | 15.535 | | 30850.773 | | 1985.841 | | Betaproteobacteriales;D_4__Burkholderiaceae;D_5__Curvibacter | |  |
| 908 | 0.002 | 0.002 | | Inf | | 5739.107 | | 0.000 | | Betaproteobacteriales;D_4__Burkholderiaceae;D_5__Delftia | |  |
| 909 | 0.009 | 0.008 | | Inf | | 1558.880 | | 0.000 | | Betaproteobacteriales;D_4__Burkholderiaceae;D_5__Diaphorobacter | |  |
| 910 | 0.013 | 0.013 | | 30.815 | | 637.724 | | 20.695 | | Betaproteobacteriales;D_4__Burkholderiaceae;D_5__Duganella | |  |
| 915 | 0.000 | 0.000 | | 44.301 | | 12774.785 | | 288.364 | | Betaproteobacteriales;D_4__Burkholderiaceae;D_5__Janthinobacterium | |  |
| 921 | 0.023 | 0.023 | | 12.854 | | 4109.775 | | 319.736 | | Betaproteobacteriales;D_4__Burkholderiaceae;D_5__Massilia | |  |
| 926 | 0.000 | 0.000 | | 139.865 | | 10593.448 | | 75.740 | | Betaproteobacteriales;D_4__Burkholderiaceae;D_5__Paucibacter | |  |
| 932 | 0.001 | 0.001 | | 44.339 | | 21081.063 | | 475.450 | | Betaproteobacteriales;D_4__Burkholderiaceae;D_5__Ralstonia | |  |
| 935 | 0.000 | 0.000 | | 14.832 | | 63620.239 | | 4289.373 | | Betaproteobacteriales;D_4__Burkholderiaceae;D_5__Rhodoferax | |  |
| 936 | 0.003 | 0.003 | | 13.413 | | 3985.681 | | 297.149 | | Betaproteobacteriales;D_4__Burkholderiaceae;D_5__Simplicispira | |  |
| 937 | 0.000 | 0.000 | | 15.560 | | 33558.273 | | 2156.665 | | Betaproteobacteriales;D_4__Burkholderiaceae;D_5__Sphaerotilus | |  |
| 949 | 0.022 | 0.022 | | 15.814 | | 567.357 | | 35.877 | | Betaproteobacteriales;D_4__Chitinimonadaceae;D_5__Chitinivorax | |  |
| 983 | 0.001 | 0.001 | | 11.225 | | 7442.507 | | 663.020 | | Betaproteobacteriales;D_4__Rhodocyclaceae;D_5__Ferribacterium | |  |
| 991 | 0.000 | 0.000 | | 10.729 | | 21237.376 | | 1979.414 | | Betaproteobacteriales;D_4__Rhodocyclaceae;__ | |  |
| 1071 | 0.000 | 0.000 | | 368.536 | | 2994070.641 | | 8124.235 | | Pseudomonadales;D_4__Moraxellaceae;D_5__Acinetobacter | |  |
| 1075 | 0.012 | 0.012 | | 33.479 | | 1586.116 | | 47.377 | | Pseudomonadales;D_4__Moraxellaceae;D_5__Perlucidibaca | |  |
| 1101 | 0.002 | 0.002 | | 47.190 | | 1079.420 | | 22.874 | | Xanthomonadales;D_4__Xanthomonadaceae;D_5__Stenotrophomonas | |  |
| 1131 | 0.009 | 0.008 | | Inf | | 212.575 | | 0.000 | | Chthoniobacterales;D_4__Chthoniobacteraceae;D_5__Chthoniobacter | |  |
|  |  |  | |  | |  | |  | |  | |  |
| 1-OCTANOL, T = 3 days | | | | |  | |  | |  | |  | |
| 63 | 0.001 | 0.001 | | 23.581 | | 8787.169 | | 372.632 | | D_3__Bifidobacteriales;D_4__Bifidobacteriaceae;D_5__Bifidobacterium | |  |
| 64 | 0.021 | 0.020 | | Inf | | 191.353 | | 0.000 | | D_3__Corynebacteriales;D_4__Corynebacteriaceae;D_5__Corynebacterium 1 | |  |
| 87 | 0.001 | 0.001 | | 22.499 | | 3110.146 | | 138.235 | | D_3__Micrococcales;D_4__Microbacteriaceae;__ | |  |
| 127 | 0.022 | 0.020 | | Inf | | 159.461 | | 0.000 | | D_3__Bacteroidales;D_4__Bacteroidetes BD2-2;__ | |  |
| 187 | 0.022 | 0.020 | | Inf | | 255.137 | | 0.000 | | D_3__Chitinophagales;D_4__Saprospiraceae;D_5__Haliscomenobacter | |  |
| 188 | 0.021 | 0.020 | | Inf | | 95.677 | | 0.000 | | D_3__Chitinophagales;D_4__Saprospiraceae;D_5__OLB8 | |  |
| 193 | 0.040 | 0.040 | | 15.501 | | 287.030 | | 18.517 | | D_3__Chitinophagales;D_4__uncultured;D_5__uncultured bacterium | |  |
| 209 | 0.041 | 0.041 | | 13.003 | | 226.620 | | 17.428 | | D_3__Cytophagales;D_4__Microscillaceae;D_5__uncultured | |  |
| 213 | 0.000 | 0.000 | | 15.455 | | 3316.101 | | 214.558 | | D_3__Cytophagales;D_4__Spirosomaceae;D_5__Emticicia | |  |
| 216 | 0.001 | 0.001 | | 18.485 | | 6622.497 | | 358.258 | | D_3__Cytophagales;D_4__Spirosomaceae;D_5__Lacihabitans | |  |
| 222 | 0.021 | 0.020 | | Inf | | 223.245 | | 0.000 | | D_3__Flavobacteriales;D_4__Crocinitomicaceae;D_5__Crocinitomix | |  |
| 223 | 0.000 | 0.000 | | 27.595 | | 34876.985 | | 1263.884 | | D_3__Flavobacteriales;D_4__Crocinitomicaceae;D_5__Fluviicola | |  |
| 229 | 0.002 | 0.002 | | 29.065 | | 117566.081 | | 4044.992 | | D_3__Flavobacteriales;D_4__Flavobacteriaceae;D_5__Flavobacterium | |  |
| 238 | 0.005 | 0.005 | | Inf | | 1223.472 | | 0.000 | | D_3__Flavobacteriales;D_4__Weeksellaceae;D_5__Elizabethkingia | |  |
| 241 | 0.021 | 0.020 | | Inf | | 382.706 | | 0.000 | | D_3__Sphingobacteriales;D_4__AKYH767;D_5__uncultured bacterium | |  |
| 244 | 0.005 | 0.005 | | 43.902 | | 962.550 | | 21.925 | | D_3__Sphingobacteriales;D_4__KD3-93;__ | |  |
| 253 | 0.021 | 0.020 | | Inf | | 97.123 | | 0.000 | | D_3__Sphingobacteriales;D_4__Sphingobacteriaceae;D_5__Mucilaginibacter | |  |
| 260 | 0.001 | 0.001 | | 24.244 | | 9744.695 | | 401.941 | | D_3__Sphingobacteriales;D_4__env.OPS 17;D_5__uncultured bacterium | |  |
| 261 | 0.001 | 0.001 | | 44.926 | | 7402.029 | | 164.759 | | D_3__Sphingobacteriales;D_4__env.OPS 17;__ | |  |
| 534 | 0.020 | 0.020 | | Inf | | 108.971 | | 0.000 | | D_3__Selenomonadales;D_4__Veillonellaceae;D_5__Megamonas | |  |
| 574 | 0.022 | 0.020 | | Inf | | 129.497 | | 0.000 | | D_3__uncultured;D_4__uncultured bacterium;D_5__uncultured bacterium | |  |
| 630 | 0.022 | 0.020 | | Inf | | 255.137 | | 0.000 | | D_3__uncultured bacterium;D_4__uncultured bacterium;D_5__uncultured bacterium | |  |
| 673 | 0.021 | 0.020 | | Inf | | 95.677 | | 0.000 | | D_3__Caulobacterales;D_4__Caulobacteraceae;D_5__Asticcacaulis | |  |
| 674 | 0.000 | 0.000 | | 20.120 | | 9359.921 | | 465.205 | | D_3__Caulobacterales;D_4__Caulobacteraceae;D_5__Brevundimonas | |  |
| 675 | 0.011 | 0.011 | | 27.344 | | 6310.957 | | 230.801 | | D_3__Caulobacterales;D_4__Caulobacteraceae;D_5__Caulobacter | |  |
| 677 | 0.022 | 0.020 | | Inf | | 574.059 | | 0.000 | | D_3__Caulobacterales;D_4__Caulobacteraceae;D_5__uncultured | |  |
| 682 | 0.021 | 0.020 | | Inf | | 223.245 | | 0.000 | | D_3__Caulobacterales;D_4__Hyphomonadaceae;D_5__uncultured | |  |
| 699 | 0.022 | 0.020 | | Inf | | 733.520 | | 0.000 | | D_3__Paracaedibacterales;D_4__Paracaedibacteraceae;D_5__uncultured | |  |
| 707 | 0.021 | 0.020 | | Inf | | 191.353 | | 0.000 | | D_3__Rhizobiales;D_4__Beijerinckiaceae;D_5__Bosea | |  |
| 715 | 0.006 | 0.006 | | Inf | | 480.793 | | 0.000 | | D_3__Rhizobiales;D_4__Devosiaceae;D_5__Devosia | |  |
| 724 | 0.003 | 0.003 | | Inf | | 1156.796 | | 0.000 | | D_3__Rhizobiales;D_4__Rhizobiaceae;D_5__Mesorhizobium | |  |
| 732 | 0.021 | 0.020 | | Inf | | 223.245 | | 0.000 | | D_3__Rhizobiales;D_4__Rhizobiales Incertae Sedis;D_5__Phreatobacter | |  |
| 745 | 0.020 | 0.020 | | Inf | | 490.371 | | 0.000 | | D_3__Rhodobacterales;D_4__Rhodobacteraceae;D_5__Rubellimicrobium | |  |
| 759 | 0.021 | 0.020 | | Inf | | 291.368 | | 0.000 | | D_3__Rickettsiales;D_4__Anaplasmataceae;D_5__Wolbachia | |  |
| 787 | 0.005 | 0.005 | | 19.423 | | 1316.257 | | 67.768 | | D_3__Sphingomonadales;D_4__Sphingomonadaceae;D_5__Sphingomonas | |  |
| 789 | 0.038 | 0.038 | | 17.181 | | 1262.596 | | 73.489 | | D_3__Sphingomonadales;D_4__Sphingomonadaceae;D_5__Sphingorhabdus | |  |
| 790 | 0.033 | 0.033 | | 23.648 | | 3699.493 | | 156.443 | | D_3__SphingomonadalesD_4__SphingomonadaceaeD_5__uncultured | |  |
| 799 | 0.022 | 0.020 | | Inf | | 159.461 | | 0.000 | | D_3__BdellovibrionalesD_4__BacteriovoracaceaeD_5__Bacteriovorax | |  |
| 808 | 0.021 | 0.020 | | Inf | | 127.569 | | 0.000 | | D_3__DesulfarculalesD_4__DesulfarculaceaeD_5__uncultured | |  |
| 837 | 0.001 | 0.001 | | 20.672 | | 19160.334 | | 926.857 | | D_3__MyxococcalesD_4__Blfdi19D_5__uncultured bacterium | |  |
| 873 | 0.020 | 0.020 | | Inf | | 194.245 | | 0.000 | | D_3__SAR324 clade(Marine group B)D_4__uncultured bacteriumD_5__uncultured bacterium | |  |
| 885 | 0.021 | 0.020 | | Inf | | 255.137 | | 0.000 | | D_2__Deltaproteobacteria____ | |  |
| 892 | 0.020 | 0.020 | | Inf | | 161.871 | | 0.000 | | D_3__AeromonadalesD_4__SuccinivibrionaceaeD_5__Succinivibrio | |  |
| 902 | 0.003 | 0.003 | | Inf | | 834.981 | | 0.000 | | D_3__BetaproteobacterialesD_4__BurkholderiaceaeD_5__Achromobacter | |  |
| 903 | 0.001 | 0.001 | | 13.820 | | 16167.975 | | 1169.919 | | D_3__BetaproteobacterialesD_4__BurkholderiaceaeD_5__Acidovorax | |  |
| 904 | 0.000 | 0.000 | | 11.104 | | 13807.536 | | 1243.469 | | D_3__BetaproteobacterialesD_4__BurkholderiaceaeD_5__Aquabacterium | |  |
| 905 | 0.001 | 0.001 | | 27.434 | | 13159.836 | | 479.695 | | D_3__BetaproteobacterialesD_4__BurkholderiaceaeD_5__Burkholderia-Caballeronia-Paraburkholderia | |  |
| 918 | 0.001 | 0.001 | | 10.443 | | 50101.818 | | 4797.448 | | D_3__BetaproteobacterialesD_4__BurkholderiaceaeD_5__Limnohabitans | |  |
| 926 | 0.008 | 0.008 | | 67.866 | | 5140.170 | | 75.740 | | D_3__BetaproteobacterialesD_4__BurkholderiaceaeD_5__Paucibacter | |  |
| 932 | 0.000 | 0.000 | | 21.509 | | 10226.580 | | 475.450 | | D_3__BetaproteobacterialesD_4__BurkholderiaceaeD_5__Ralstonia | |  |
| 960 | 0.042 | 0.042 | | 10.954 | | 938.853 | | 85.706 | | D_3__BetaproteobacterialesD_4__MethylophilaceaeD_5__Methylobacillus | |  |
| 970 | 0.021 | 0.020 | | Inf | | 318.922 | | 0.000 | | D_3__BetaproteobacterialesD_4__NitrosomonadaceaeD_5__MND1 | |  |
| 1071 | 0.001 | 0.001 | | 206.311 | | 1676118.210 | | 8124.235 | | D_3__PseudomonadalesD_4__MoraxellaceaeD_5__Acinetobacter | |  |
| 1077 | 0.000 | 0.000 | | 14.162 | | 2928.435 | | 206.783 | | D_3__PseudomonadalesD_4__MoraxellaceaeD_5__uncultured | |  |
| 1079 | 0.000 | 0.000 | | 29.187 | | 901402.594 | | 30883.318 | | D_3__PseudomonadalesD_4__PseudomonadaceaeD_5__Pseudomonas | |  |
| 1101 | 0.003 | 0.003 | | 146.057 | | 3340.890 | | 22.874 | | D_3__XanthomonadalesD_4__XanthomonadaceaeD_5__Stenotrophomonas | |  |
| 1141 | 0.042 | 0.042 | | 10.350 | | 381.399 | | 36.849 | | D_3__OpitutalesD_4__OpitutaceaeD_5__Opitutus | |  |
|  |  |  | |  | |  | |  | |  | |  |
| METHYLJASMONATE, T = 3 days | | | | |  | |  | |  | |  | |
| 70 | 0.017 | 0.015 | | Inf | | 29.148 | | 0 | | Frankiales;D_4__Nakamurellaceae;D_5__Nakamurella | |  |
| 211 | 0.007 | 0.007 | | 20.661 | | 3030.954 | | 146.698 | | Cytophagales;D_4__Spirosomaceae;D_5__Arcicella | |  |
| 240 | 0.004 | 0.004 | | 14.202 | | 1305.622 | | 91.929 | | Flavobacteriales;__;__ | |  |
| 379 | 0.017 | 0.015 | | Inf | | 58.502 | | 0 | | Deinococcales;D_4__Trueperaceae;D_5__Truepera | |  |
| 610 | 0.016 | 0.015 | | Inf | | 46.74 | | 0 | | Candidatus Moranbacteria;D_4__uncultured bacterium;D_5__uncultured bacterium | |  |
| 675 | 0 | 0 | | 16.086 | | 3712.617 | | 230.801 | | Caulobacterales;D_4__Caulobacteraceae;D_5__Caulobacter | |  |
| 781 | 0.001 | 0.001 | | 77.648 | | 10989.121 | | 141.524 | | Sphingomonadales;D_4__Sphingomonadaceae;D_5__Novosphingobium | |  |
| 786 | 0.001 | 0.001 | | 219.07 | | 30612.283 | | 139.738 | | Sphingomonadales;D_4__Sphingomonadaceae;D_5__Sphingobium | |  |
| 819 | 0.016 | 0.015 | | Inf | | 17.489 | | 0 | | Desulfobacterales;D_4__Desulfobulbaceae;D_5__[Desulfobacterium] catecholicum group | |  |
| 849 | 0.009 | 0.009 | | 10.044 | | 6259.749 | | 623.26 | | Myxococcales;D_4__Polyangiaceae;D_5__Pajaroellobacter | |  |
| 903 | 0.001 | 0.001 | | 26.826 | | 31384.066 | | 1169.919 | | Betaproteobacteriales;D_4__Burkholderiaceae;D_5__Acidovorax | |  |
| 910 | 0.002 | 0.002 | | 115.506 | | 2390.443 | | 20.695 | | Betaproteobacteriales;D_4__Burkholderiaceae;D_5__Duganella | |  |
| 912 | 0.002 | 0.002 | | 13.79 | | 41964.518 | | 3043.011 | | Betaproteobacteriales;D_4__Burkholderiaceae;D_5__Hydrogenophaga | |  |
| 913 | 0.004 | 0.004 | | Inf | | 2054.737 | | 0 | | Betaproteobacteriales;D_4__Burkholderiaceae;D_5__Ideonella | |  |
| 960 | 0.007 | 0.007 | | 14.5 | | 1242.778 | | 85.706 | | Betaproteobacteriales;D_4__Methylophilaceae;D_5__Methylobacillus | |  |
| 1004 | 0.016 | 0.015 | | Inf | | 1004.916 | | 0 | | Cellvibrionales;D_4__Cellvibrionaceae;D_5__Aestuariicella | |  |
| 1010 | 0.003 | 0.003 | | Inf | | 3055.541 | | 0 | | Cellvibrionales;D_4__Cellvibrionaceae;__ | |  |
| 1071 | 0.012 | 0.012 | | 14.352 | | 116597.609 | | 8124.235 | | Pseudomonadales;D_4__Moraxellaceae;D_5__Acinetobacter | |  |
|  |  |  | |  | |  | |  | |  | |  |
| METHYLJASMONATE, T = 6 days | | | | |  | |  | |  | |  | |
| 80 | 0.021 | 0.02 | | Inf | | 403.938 | | 0 | | Micrococcales;D_4__Microbacteriaceae;D_5__Candidatus Aquiluna | |  |
| 87 | 0.039 | 0.039 | | 18.715 | | 1341.075 | | 71.657 | | Micrococcales;D_4__Microbacteriaceae;__ | |  |
| 183 | 0.04 | 0.04 | | 10.318 | | 177.733 | | 17.225 | | Chitinophagales;D_4__Chitinophagaceae;D_5__uncultured | |  |
| 211 | 0.021 | 0.02 | | Inf | | 5655.133 | | 0 | | Cytophagales;D_4__Spirosomaceae;D_5__Arcicella | |  |
| 213 | 0.021 | 0.02 | | Inf | | 323.15 | | 0 | | Cytophagales;D_4__Spirosomaceae;D_5__Emticicia | |  |
| 216 | 0.02 | 0.02 | | Inf | | 1761.17 | | 0 | | Cytophagales;D_4__Spirosomaceae;D_5__Lacihabitans | |  |
| 220 | 0.021 | 0.02 | | Inf | | 258.52 | | 0 | | Cytophagales;D_4__Spirosomaceae;__ | |  |
| 226 | 0.021 | 0.02 | | Inf | | 398.456 | | 0 | | Flavobacteriales;D_4__Cryomorphaceae;D_5__NS10 marine group | |  |
| 227 | 0.003 | 0.003 | | Inf | | 289.213 | | 0 | | Flavobacteriales;D_4__Cryomorphaceae;D_5__uncultured | |  |
| 229 | 0 | 0 | | 13.743 | | 69499.771 | | 5057.082 | | Flavobacteriales;D_4__Flavobacteriaceae;D_5__Flavobacterium | |  |
| 236 | 0.021 | 0.02 | | Inf | | 312.788 | | 0 | | Flavobacteriales;D_4__Weeksellaceae;D_5__Chryseobacterium | |  |
| 240 | 0.004 | 0.004 | | 44.164 | | 3803.702 | | 86.127 | | Flavobacteriales;__;__ | |  |
| 250 | 0.004 | 0.004 | | 20.539 | | 3789.897 | | 184.526 | | Sphingobacteriales;D_4__NS11-12 marine group;D_5__uncultured bacterium | |  |
| 284 | 0.02 | 0.02 | | Inf | | 351.887 | | 0 | | Chlamydiales;D_4__Parachlamydiaceae;__ | |  |
| 369 | 0.02 | 0.02 | | Inf | | 265.637 | | 0 | | Pseudanabaenales;D_4__Pseudanabaenaceae;D_5__Pseudanabaena PCC-7429 | |  |
| 375 | 0 | 0 | | 12.974 | | 3857.543 | | 297.338 | | uncultured bacterium;D_4__uncultured bacterium;D_5__uncultured bacterium | |  |
| 630 | 0.02 | 0.02 | | Inf | | 96.945 | | 0 | | uncultured bacterium;D_4__uncultured bacterium;D_5__uncultured bacterium | |  |
| 672 | 0.009 | 0.009 | | 23.669 | | 1538.169 | | 64.987 | | Caedibacterales;D_4__Caedibacteraceae;D_5__Caedibacter | |  |
| 694 | 0.021 | 0.02 | | Inf | | 145.418 | | 0 | | Micropepsales;D_4__Micropepsaceae;D_5__uncultured | |  |
| 700 | 0.02 | 0.02 | | Inf | | 80.788 | | 0 | | Paracaedibacterales;D_4__Paracaedibacteraceae;__ | |  |
| 715 | 0.02 | 0.02 | | Inf | | 533.198 | | 0 | | Rhizobiales;D_4__Devosiaceae;D_5__Devosia | |  |
| 716 | 0.02 | 0.02 | | Inf | | 796.912 | | 0 | | Rhizobiales;D_4__Hyphomicrobiaceae;D_5__Hyphomicrobium | |  |
| 767 | 0.02 | 0.02 | | Inf | | 80.788 | | 0 | | Rickettsiales;D_4__Rickettsiaceae;D_5__Candidatus Megaira | |  |
| 778 | 0.02 | 0.02 | | Inf | | 64.63 | | 0 | | Sneathiellales;D_4__Sneathiellaceae;D_5__Taonella | |  |
| 781 | 0.002 | 0.002 | | 278.653 | | 63378.908 | | 227.448 | | Sphingomonadales;D_4__Sphingomonadaceae;D_5__Novosphingobium | |  |
| 786 | 0.004 | 0.004 | | 2432.05 | | 120171.532 | | 49.412 | | Sphingomonadales;D_4__Sphingomonadaceae;D_5__Sphingobium | |  |
| 793 | 0.02 | 0.02 | | Inf | | 96.945 | | 0 | | Tistrellales;D_4__Geminicoccaceae;D_5__Candidatus Alysiosphaera | |  |
| 798 | 0.02 | 0.02 | | Inf | | 64.63 | | 0 | | D_2__Alphaproteobacteria;__;__;__ | |  |
| 804 | 0.021 | 0.02 | | Inf | | 177.733 | | 0 | | Bdellovibrionales;D_4__Bdellovibrionaceae;D_5__OM27 clade | |  |
| 837 | 0.006 | 0.006 | | 21.295 | | 6111.143 | | 286.978 | | Myxococcales;D_4__Blfdi19;D_5__uncultured bacterium | |  |
| 840 | 0.02 | 0.02 | | Inf | | 129.26 | | 0 | | Myxococcales;D_4__Haliangiaceae;D_5__Haliangium | |  |
| 849 | 0.004 | 0.004 | | 15.93 | | 6366.17 | | 399.623 | | Myxococcales;D_4__Polyangiaceae;D_5__Pajaroellobacter | |  |
| 850 | 0.001 | 0.001 | | 17.242 | | 2847.358 | | 165.144 | | Myxococcales;D_4__Sandaracinaceae;D_5__Sandaracinus | |  |
| 859 | 0.021 | 0.02 | | Inf | | 290.835 | | 0 | | Myxococcales;D_4__mle1-27;__ | |  |
| 875 | 0.02 | 0.02 | | Inf | | 199.228 | | 0 | | Sva0485;D_4__uncultured bacterium;D_5__uncultured bacterium | |  |
| 894 | 0.02 | 0.02 | | Inf | | 664.093 | | 0 | | Alteromonadales;D_4__Shewanellaceae;D_5__Shewanella | |  |
| 903 | 0.001 | 0.001 | | 68.15 | | 133161.523 | | 1953.938 | | Betaproteobacteriales;D_4__Burkholderiaceae;D_5__Acidovorax | |  |
| 907 | 0.001 | 0.001 | | 24.388 | | 130794.311 | | 5362.978 | | Betaproteobacteriales;D_4__Burkholderiaceae;D_5__Curvibacter | |  |
| 910 | 0.02 | 0.02 | | Inf | | 436.253 | | 0 | | Betaproteobacteriales;D_4__Burkholderiaceae;D_5__Duganella | |  |
| 911 | 0 | 0 | | 72.967 | | 2970.823 | | 40.715 | | Betaproteobacteriales;D_4__Burkholderiaceae;D_5__GKS98 freshwater group | |  |
| 912 | 0.001 | 0.001 | | 48.939 | | 116350.003 | | 2377.465 | | Betaproteobacteriales;D_4__Burkholderiaceae;D_5__Hydrogenophaga | |  |
| 921 | 0.029 | 0.029 | | 76.117 | | 4648.653 | | 61.072 | | Betaproteobacteriales;D_4__Burkholderiaceae;D_5__Massilia | |  |
| 929 | 0.002 | 0.002 | | 22.44 | | 36049.22 | | 1606.473 | | Betaproteobacteriales;D_4__Burkholderiaceae;D_5__Polaromonas | |  |
| 932 | 0 | 0 | | 13.569 | | 10610.989 | | 781.991 | | Betaproteobacteriales;D_4__Burkholderiaceae;D_5__Ralstonia | |  |
| 939 | 0.021 | 0.02 | | Inf | | 821.069 | | 0 | | Betaproteobacteriales;D_4__Burkholderiaceae;D_5__Tepidimonas | |  |
| 959 | 0.016 | 0.016 | | 11.786 | | 5332.607 | | 452.44 | | Betaproteobacteriales;D_4__Methylophilaceae;D_5__Candidatus Methylopumilus | |  |
| 961 | 0.002 | 0.002 | | 24.067 | | 5839.446 | | 242.634 | | Betaproteobacteriales;D_4__Methylophilaceae;D_5__Methylophilus | |  |
| 962 | 0.004 | 0.004 | | 54.354 | | 147231.884 | | 2708.765 | | Betaproteobacteriales;D_4__Methylophilaceae;D_5__Methylotenera | |  |
| 963 | 0.011 | 0.011 | | 28.066 | | 9273.946 | | 330.438 | | Betaproteobacteriales;D_4__Methylophilaceae;__ | |  |
| 966 | 0.04 | 0.04 | | 10.3 | | 630.143 | | 61.176 | | Betaproteobacteriales;D_4__Neisseriaceae;D_5__uncultured | |  |
| 1006 | 0.02 | 0.02 | | Inf | | 678.616 | | 0 | | Cellvibrionales;D_4__Cellvibrionaceae;D_5__Cellvibrio | |  |
| 1028 | 0.02 | 0.02 | | Inf | | 531.275 | | 0 | | Ectothiorhodospirales;D_4__Thioalkalispiraceae;D_5__Thioalkalispira | |  |
| 1048 | 0.02 | 0.02 | | Inf | | 161.575 | | 0 | | KI89A clade;__;__ | |  |
| 1100 | 0.039 | 0.039 | | 20.286 | | 508.281 | | 25.055 | | Xanthomonadales;D_4__Xanthomonadaceae;D_5__Pseudoxanthomonas | |  |
| 1106 | 0.007 | 0.007 | | 10.173 | | 2848.712 | | 280.025 | | Gammaproteobacteria;D_3__uncultured;__;__ | |  |
| 1160 | 0.021 | 0.02 | | Inf | | 156.394 | | 0 | | Verrucomicrobiae;D_3__uncultured;__;__ | |  |
| 1163 | 0.006 | 0.006 | | 10.53 | | 538.994 | | 51.185 | | D_1__WPS-2;D_2__uncultured bacterium;D_3__uncultured bacterium;D_4__uncultured bacterium;D_5__uncultured bacterium | |  |
|  |  |  | |  | |  | |  | |  | |  |
|  |  |  | |  | |  | |  | |  | |  |
| MYRCENE, T = 3 days | | |  | |  | |  | |  | |  | |
| 80 | 0.028 | 0.028 | | Inf | | 43.617 | | 0 | | Micrococcales;D_4__Microbacteriaceae;D_5__Candidatus Aquiluna | |  |
| 83 | 0.028 | 0.028 | | Inf | | 61.626 | | 0 | | Micrococcales;D_4__Microbacteriaceae;D_5__Leucobacter | |  |
| 184 | 0.03 | 0.028 | | Inf | | 90.466 | | 0 | | Chitinophagales;D_4__Chitinophagaceae;__ | |  |
| 187 | 0.028 | 0.028 | | Inf | | 29.078 | | 0 | | Chitinophagales;D_4__Saprospiraceae;D_5__Haliscomenobacter | |  |
| 211 | 0.03 | 0.028 | | Inf | | 48.463 | | 0 | | Cytophagales;D_4__Spirosomaceae;D_5__Arcicella | |  |
| 213 | 0.005 | 0.005 | | 14.745 | | 2163.063 | | 146.698 | | Cytophagales;D_4__Spirosomaceae;D_5__Emticicia | |  |
| 262 | 0.003 | 0.003 | | 12.653 | | 2714.908 | | 214.558 | | Sphingobacteriales;__;__ | |  |
| 338 | 0.049 | 0.049 | | 19.124 | | 381.169 | | 19.932 | | Chloroplast;D_4__uncultured bacterium;D_5__uncultured bacterium | |  |
| 362 | 0.03 | 0.028 | | Inf | | 169.621 | | 0 | | Nostocales;__;__ | |  |
| 557 | 0.029 | 0.028 | | Inf | | 72.695 | | 0 | | WCHB1-41;__;__ | |  |
| 665 | 0.03 | 0.028 | | Inf | | 24.232 | | 0 | | Acetobacterales;D_4__Acetobacteraceae;D_5__Rhodovastum | |  |
| 727 | 0.028 | 0.028 | | Inf | | 120.622 | | 0 | | Rhizobiales;D_4__Rhizobiaceae;D_5__Shinella | |  |
| 735 | 0.03 | 0.028 | | Inf | | 116.405 | | 0 | | Rhizobiales;D_4__Xanthobacteraceae;D_5__Afipia | |  |
| 781 | 0.03 | 0.028 | | Inf | | 38.77 | | 0 | | Sphingomonadales;D_4__Sphingomonadaceae;D_5__Novosphingobium | |  |
| 784 | 0.01 | 0.01 | | 16.768 | | 2373.15 | | 141.524 | | Sphingomonadales;D_4__Sphingomonadaceae;D_5__Rhizorhapis | |  |
| 786 | 0.029 | 0.028 | | Inf | | 159.928 | | 0 | | Sphingomonadales;D_4__Sphingomonadaceae;D_5__Sphingobium | |  |
| 810 | 0.022 | 0.022 | | 10.424 | | 1456.655 | | 139.738 | | Desulfobacterales;D_4__Desulfobacteraceae;D_5__Desulfatirhabdium | |  |
| 913 | 0.011 | 0.011 | | 13.055 | | 219.917 | | 16.845 | | Betaproteobacteriales;D_4__Burkholderiaceae;D_5__Ideonella | |  |
| 980 | 0.001 | 0.001 | | Inf | | 836.551 | | 0 | | Betaproteobacteriales;D_4__Rhodocyclaceae;D_5__Dechlorobacter | |  |
| 1004 | 0.029 | 0.028 | | Inf | | 36.519 | | 0 | | Cellvibrionales;D_4__Cellvibrionaceae;D_5__Aestuariicella | |  |
| 1010 | 0 | 0 | | Inf | | 896.925 | | 0 | | Cellvibrionales;D_4__Cellvibrionaceae;__ | |  |
| 1100 | 0.002 | 0.002 | | Inf | | 1463.571 | | 0 | | Xanthomonadales;D_4__Xanthomonadaceae;D_5__Pseudoxanthomonas | |  |
| 1153 | 0.01 | 0.01 | | Inf | | 149.807 | | 0 | | Verrucomicrobiales;D_4__Rubritaleaceae;D_5__Luteolibacter | |  |
| 1160 | 0.055 | 0.055 | | 13.59 | | 300.471 | | 22.109 | | Verrucomicrobiae;D_3__uncultured;__;__ | |  |
|  |  |  | |  | |  | |  | |  | |  |
| MYRCENE, T = 6 days | | |  | |  | |  | |  | |  | |
| 27 | 0.045 | 0.042 | | Inf | | 21.404 | | 0 | | Holophagales;D_4__Holophagaceae;D_5__uncultured | |  |
| 94 | 0.045 | 0.042 | | Inf | | 42.808 | | 0 | | Propionibacteriales;D_4__Nocardioidaceae;D_5__Aeromicrobium | |  |
| 187 | 0.045 | 0.042 | | Inf | | 17.947 | | 0 | | Chitinophagales;D_4__Saprospiraceae;D_5__Haliscomenobacter | |  |
| 211 | 0.045 | 0.042 | | Inf | | 215.365 | | 0 | | Cytophagales;D_4__Spirosomaceae;D_5__Arcicella | |  |
| 213 | 0.043 | 0.042 | | Inf | | 38.138 | | 0 | | Cytophagales;D_4__Spirosomaceae;D_5__Emticicia | |  |
| 216 | 0.024 | 0.024 | | Inf | | 252.757 | | 0 | | Cytophagales;D_4__Spirosomaceae;D_5__Lacihabitans | |  |
| 238 | 0.043 | 0.042 | | Inf | | 73.941 | | 0 | | Flavobacteriales;D_4__Weeksellaceae;D_5__Elizabethkingia | |  |
| 244 | 0.043 | 0.042 | | Inf | | 24.739 | | 0 | | Sphingobacteriales;D_4__KD3-93;__ | |  |
| 323 | 0.044 | 0.042 | | Inf | | 22.434 | | 0 | | D_2__KD4-96;__;__;__ | |  |
| 372 | 0.043 | 0.042 | | Inf | | 82.463 | | 0 | | Synechococcales;D_4__Cyanobiaceae;D_5__Cyanobium PCC-6307 | |  |
| 414 | 0.043 | 0.042 | | Inf | | 20.191 | | 0 | | Fibrobacterales;D_4__Fibrobacteraceae;D_5__possible genus 04 | |  |
| 441 | 0.043 | 0.042 | | Inf | | 23.365 | | 0 | | Clostridiales;D_4__Clostridiaceae 1;D_5__Clostridium sensu stricto 12 | |  |
| 536 | 0.043 | 0.042 | | Inf | | 40.381 | | 0 | | Selenomonadales;D_4__Veillonellaceae;D_5__Pelosinus | |  |
| 603 | 0.044 | 0.042 | | Inf | | 26.113 | | 0 | | D_2__Gracilibacteria;__;__;__ | |  |
| 664 | 0.044 | 0.042 | | Inf | | 47.111 | | 0 | | Acetobacterales;D_4__Acetobacteraceae;D_5__Acidocella | |  |
| 708 | 0.043 | 0.042 | | Inf | | 20.191 | | 0 | | Rhizobiales;D_4__Beijerinckiaceae;D_5__Camelimonas | |  |
| 716 | 0.044 | 0.042 | | Inf | | 42.624 | | 0 | | Rhizobiales;D_4__Hyphomicrobiaceae;D_5__Hyphomicrobium | |  |
| 724 | 0.045 | 0.042 | | Inf | | 77.833 | | 0 | | Rhizobiales;D_4__Rhizobiaceae;D_5__Mesorhizobium | |  |
| 767 | 0.044 | 0.042 | | Inf | | 22.434 | | 0 | | Rickettsiales;D_4__Rickettsiaceae;D_5__Candidatus Megaira | |  |
| 786 | 0.03 | 0.03 | | 38.177 | | 1886.367 | | 49.412 | | Sphingomonadales;D_4__Sphingomonadaceae;D_5__Sphingobium | |  |
| 787 | 0.013 | 0.013 | | Inf | | 75.388 | | 0 | | Sphingomonadales;D_4__Sphingomonadaceae;D_5__Sphingomonas | |  |
| 788 | 0.045 | 0.042 | | Inf | | 60.572 | | 0 | | Sphingomonadales;D_4__Sphingomonadaceae;D_5__Sphingopyxis | |  |
| 793 | 0.044 | 0.042 | | Inf | | 24.677 | | 0 | | Tistrellales;D_4__Geminicoccaceae;D_5__Candidatus Alysiosphaera | |  |
| 840 | 0.043 | 0.042 | | Inf | | 40.381 | | 0 | | Myxococcales;D_4__Haliangiaceae;D_5__Haliangium | |  |
| 848 | 0.044 | 0.042 | | Inf | | 42.624 | | 0 | | Myxococcales;D_4__Phaselicystidaceae;D_5__Phaselicystis | |  |
| 873 | 0.01 | 0.01 | | Inf | | 39.937 | | 0 | | SAR324 clade(Marine group B);D_4__uncultured bacterium;D_5__uncultured bacterium | |  |
| 901 | 0.045 | 0.042 | | Inf | | 29.164 | | 0 | | Betaproteobacteriales;D_4__Burkholderiaceae;D_5__AAP99 | |  |
| 906 | 0.044 | 0.042 | | Inf | | 28.862 | | 0 | | Betaproteobacteriales;D_4__Burkholderiaceae;D_5__Comamonas | |  |
| 910 | 0.045 | 0.042 | | Inf | | 243.266 | | 0 | | Betaproteobacteriales;D_4__Burkholderiaceae;D_5__Duganella | |  |
| 913 | 0.044 | 0.042 | | Inf | | 170.498 | | 0 | | Betaproteobacteriales;D_4__Burkholderiaceae;D_5__Ideonella | |  |
| 931 | 0.044 | 0.042 | | Inf | | 51.598 | | 0 | | Betaproteobacteriales;D_4__Burkholderiaceae;D_5__Pseudorhodoferax | |  |
| 942 | 0.029 | 0.029 | | Inf | | 235.759 | | 0 | | Betaproteobacteriales;D_4__Burkholderiaceae;D_5__Verticia | |  |
| 978 | 0.044 | 0.042 | | Inf | | 42.624 | | 0 | | Betaproteobacteriales;D_4__Rhodocyclaceae;D_5__C39 | |  |
| 982 | 0.043 | 0.042 | | Inf | | 29.187 | | 0 | | Betaproteobacteriales;D_4__Rhodocyclaceae;D_5__Denitratisoma | |  |
| 984 | 0.043 | 0.042 | | Inf | | 50.591 | | 0 | | Betaproteobacteriales;D_4__Rhodocyclaceae;D_5__Methyloversatilis | |  |
| 1004 | 0.008 | 0.008 | | Inf | | 115.547 | | 0 | | Cellvibrionales;D_4__Cellvibrionaceae;D_5__Aestuariicella | |  |
| 1006 | 0.043 | 0.042 | | Inf | | 25.296 | | 0 | | Cellvibrionales;D_4__Cellvibrionaceae;D_5__Cellvibrio | |  |
| 1010 | 0.013 | 0.013 | | Inf | | 432.173 | | 0 | | Cellvibrionales;D_4__Cellvibrionaceae;__ | |  |
| 1019 | 0.043 | 0.042 | | Inf | | 24.677 | | 0 | | Competibacterales;D_4__Competibacteraceae;D_5__Candidatus Competibacter | |  |
| 1072 | 0.042 | 0.042 | | Inf | | 17.512 | | 0 | | Pseudomonadales;D_4__Moraxellaceae;D_5__Alkanindiges | |  |
| 1153 | 0.012 | 0.012 | | Inf | | 90.364 | | 0 | | Verrucomicrobiales;D_4__Rubritaleaceae;D_5__Luteolibacter | |  |
| 1160 | 0.042 | 0.042 | | Inf | | 19.241 | | 0 | | Verrucomicrobiae;D_3__uncultured;__;__ | |  |
