# Supplementary material for: Assessing Biodegradability of Chemical Compounds from Microbial Community Growth Using Flow Cytometry
Source: mSystems. 2021 Feb 9;6(1):e01143-20. doi: 10.1128/mSystems.01143-20 (PMC7883543; doi:10.1128/mSystems.01143-20)
Supplement: TABLE S4 [file mSystems.01143-20-st004.docx]

| **Chemical name** | **CAS No** | **Chemical formula** | **Mol. Weight (g mol^-1^)** | **Carbon mol weight (g mol^-1^)** | **Water sol.**  **(mg l^-1^)** | **Henry’s Law Constant (atm-m^3^ mol^_1^)** | **Log K_ow_** | **Log K_aw_** | **Log K_oa_** | **Biodegradability** |
| --- | --- | --- | --- | --- | --- | --- | --- | --- | --- | --- |
| 1-octanol | (111-87-5) | C_8_H_18_O | 130.23 | 96 | 814 | 3·10^–5^ | 2.81 | –2.99 | 5.70 | readily |
| Phenol | (108-95-2) | C_6_H_6_O | 94.11 | 72 | 46000 | 3.3·10^–7^ | 1.46 | –4.87 | 6.33 | readily |
| Sodium benzoate | (532-32-1) | C_7_H_5_O_2_Na | 144.11 | 84 | 1.0·10^6^ | 1.09·10^–7^ | –2.27 | –5.35 | 3.08 | readily |
| Methyl Jasmonate | (1211-29-6) | C_13_H_20_O_3_ | 224.30 | 156 | 143.5 | 6.93·10^–7^ | 2.76 | –4.74 | 7.50 | intermediate |
| Myrcene | (123-35-3) | C_10_H_16_ | 136.24 | 120 | 6.92 | 0.064 | 4.88 | 0.42 | 3.75 | intermediate |
| Musk xylene | (81-15-2) | C_12_H_15_N_3_O_6_ | 297.27 | 144 | 0.15 | 7.73·10^–9^ | 4.4 | NA | NA | non-biodegradable |

a) Properties according to the US-EPA, Estimation Program Interface (EPI) Suite. Ver. 4.
